# What Has Happened Since the Implementation of the Clinical Trials Act? Epidemiologists Need to Know

**DOI:** 10.2188/jea.JE20210425

**Published:** 2022-01-05

**Authors:** Yuri Ito, Seiichiro Yamamoto, Kenichi Nakamura

**Affiliations:** 1 Osaka Medical and Pharmaceutical University, Osaka, Japan; 2 Shizuoka Graduate University of Public Health, Shizuoka, Japan; 3 National Cancer Center Institute for Cancer Control, Tokyo, Japan; 4 National Cancer Center Hospital, Tokyo, Japan

Tsutsumi et al have reported a decrease in the number of clinical trials in Japan following implementation of the Clinical Trials Act (CTA) in 2018.^1^ The CTA was introduced to improve the quality and transparency of clinical trials in Japan. However, the installation of the new legislation—especially administrative processes, such as verification of conflict of interests, submission of the trial plan to the government, and increased review fees for review board certification—is extremely burdensome. As a result, some research activities in Japan may have been suppressed.^2^

Tsutsumi et al collected data on clinical studies using the University hospital Medical Information Network Clinical Trials Registry (UMIN-CTR) and analyzed 16,455 studies which started between April 2015 and March 2019. The dataset included clinical studies started 1 year after implementation of the CTA (April 2018 to March 2019). They analyzed trends in the number of clinical trials per month using interrupted time-series analysis (ITSA). They observed a significantly decreasing trend in the year following CTA implementation. In particular, studies with smaller sample sizes (*n* < 100), interventional designs, and non-profit funding sponsors decreased considerably. Although it is very important to improve quality and transparency of clinical studies in Japan, there is a serious problem if research activities in Japan have been suppressed by the new legislation. This high impact result showed the need for support of clinical studies in Japan.

Although the authors did not suggest this in the article, the decreasing trend of new clinical studies after the CTA implementation might be strongly related to the increased workload for the reauthorization of studies that had started before the act, as a transitional measure of the CTA. Research institutes with a larger number of studies might have faced an onerous and time-consuming task in completing reauthorization for the certification review board (CRB).

According to a survey of the Japanese Cancer Trial Network (JCTN), which is a consortium of large clinical trial groups in Japan, the number of clinical trials JCTN conducted increased after FY2019, although the number had decreased once in FY2018, just after the CTA implementation.^3^ The research facilities in the JCTN have adapted to the CTA after 1 year but with a significant cost, especially in terms of increased expenditure, staff costs, and difficulties of complicated administration for the support section and researchers. According to a survey by Kunito et al, about 80% of researchers reported that the burden of the CTA implementation includes an increase in the cost of the CRB and insurance for clinical trials, as well as complicated administrative processes, including setup for new research and small changes to protocols. In addition, over 80% complained about the discrepancy in interpretation of the usage and dosage of medication between the clinical and the research setting under the CTA.^4^

Research institutions and hospitals in the JCTN might have quickly adapted to the CTA with 1 year after implementation. However, many researchers and institutions with insufficient supporting resources might still have struggled with the burden of cost and complicated administration. We need to consider the problem in order to improve the situation and boost clinical research activities in Japan. For example, leading groups, such as the JCTN, have accumulated tips on how to deal with the CTA and started to share effective strategies with other research institution.^5^^,^^6^ As Tsutsumi et al also suggested, further middle- or long-term monitoring is needed after 2020 using public data from UMIN-CTR and the Japan Registry for Clinical Trials. In addition, we need to be conscious of inequalities in research environments among research institutions regarding financial and human resources.

Revision of the CTA has also been proposed by several research associations, such as JCTN,^7^ the Japanese Medical Science Federation,^8^ and the Japan Society of Clinical Trials and Research, which support clinical studies in academic settings in order to reduce complicated processes with reliability and transparency of clinical studies.^7^ Revision of the CTA is required to clarify some points, such as showing a clear distinction between the clinical research covered by the CTA and the ethical guidelines for other interventional and observational research. Ideally, if the intervention trials were conducted following the CTA rules, evidence from trials of sufficient quality could be used for the process of authorization and/or indication expansion of drugs. Amendment of the CTA is now under discussion in a committee of the Ministry of Health, Labour and Welfare. The CTA is to be amended within 5 years of implementation; reasonable revisions are expected to be made to revitalize the clinical trial activity, keeping the trial quality and subject safety sound.

Another issue of the CTA is the lack of international consistency. When international clinical trials are conducted under the CTA, Japanese investigators necessarily confront several difficulties in harmonizing the CTA with the regulation policies of other countries. For example, the unclear scope of the CTA, absence of the concept of sponsorship, extreme regulation regarding the development of medical devices, the unique rule of serious adverse event reporting, and overly strict conflict of interest management differ from international standards. In light of the COVID-19 situation, we should not just push forward considering Japan in isolation but should take it in the direction of international consistency and international joint development of new medicines and devices.

## IMPLICATION FOR EPIDEMIOLOGISTS

In Japan, there are still few interventional trials in the field of epidemiology, and the use of observational studies is widespread. Although it is very important to reveal the relationships between exposures and outcomes using observational data, we can move on to the next step of developing preventive strategies and effective behavioral changes using the interventional study approach after the identification of risk factors. In addition, a sizeable number of members often support clinical studies as an epidemiologist or biostatistician. Therefore, epidemiologists need to know the basic methods and rules of interventional studies. We also need to be aware of the influence of the implementation of the CTA, including the issues and points that need to be revised. We hope that our epidemiologists can understand the situation and boost the possibility of conducting more interventional studies in the field of epidemiology in Japan.
